# Inflamed macrophage microvesicles induce insulin resistance in human adipocytes

**DOI:** 10.1186/s12986-015-0016-3

**Published:** 2015-06-06

**Authors:** Yaqin Zhang, Li Shi, Hongliang Mei, Jiexin Zhang, Yunxia Zhu, Xiao Han, Dalong Zhu

**Affiliations:** The Affiliated Drum Tower Hospital of Nanjing Medical University, Nanjing, Jiangsu 21008 China; Department of Biochemistry and Molecular Biology, Key Laboratory of Human Functional Genomics of Jiangsu Province, Nanjing Medical University, Nanjing, Jiangsu 210029 China; Department of Laboratory Medicine, the First Affiliated Hospital of Nanjing Medical University, Nanjing, Jiangsu 210029 China

**Keywords:** Insulin resistance, Human adipocyte, Macrophage, Microvesicle

## Abstract

**Background:**

Cytokines secreted by adipose tissue macrophages (ATMs) significantly alter adipocyte function, inducing inflammatory responses and decreasing insulin sensitivity. However, little relevant information is available regarding the role of microvesicles (MVs) derived from ATMs in macrophage-adipocyte crosstalk.

**Methods:**

MVs were generated by stimulation of M1 or M2 phenotype THP-1 macrophages and incubated with human primary mature adipocytes and differentiated adipocytes. Subsequently, insulin-stimulated phosphorylation of Akt (pAkt) and glucose uptake were determined. Glucose transporter 4 (GLUT4) translocation and nuclear translocation of nuclear factor (NF)-kappa B were also analyzed in treated adipocytes.

**Results:**

M1 macrophage-derived MVs (M1 MVs) significantly reduced protein abundance of insulin-induced Akt phosphorylation in human primary mature adipocytes and differentiated adipocytes, when compared with the same concentration of M2 macrophage-derived MVs (M2 MVs). In contrast to M2 MVs, which enhanced the insulin-induced glucose uptake measured by 2-NBDG, M1 MVs decreased this effect in treated adipocytes. M1 MVs treatment also brought about a significant increase in the nuclear translocation of nuclear factor (NF)-kappa B, coupled with a decrease in pAkt level and GLUT4 translocation compared with M2 MVs-treated adipocytes. These effects were reversed by BAY 11–7085, a NF- kappa B specific inhibitor.

**Conclusions:**

MVs derived from proinflammatory (M1) macrophages may, at least in part, contribute to the pathogenesis of obesity-induced insulin resistance, reducing insulin signal transduction and decreasing glucose uptake in human adipocytes, through NF-kappa B activation. Therefore, these MVs may be potential therapy candidates for the management of type 2 diabetes mellitus.

**Electronic supplementary material:**

The online version of this article (doi:10.1186/s12986-015-0016-3) contains supplementary material, which is available to authorized users.

## Background

Obesity-induced insulin resistance is a key component in the pathogenesis of type 2 diabetes (T2D) [1]. Adipose tissue macrophages (ATMs) are necessary and sufficient for the development of the adipose tissue (AT) inflammation and insulin resistance associated with obesity [2]. The general understanding is that ATMs are mainly subdivided into M1 (classically activated or pro-inflammatory) and M2 (alternatively activated or anti-inflammatory) phenotypes, wherein M1 ATMs show a pro-inflammatory character, whereas M2 ATMs exhibit an anti-inflammatory phenotype [3, 4]. Increasing evidence has demonstrated that the cytokines [e.g., tumor necrosis factor-α (TNF-α) [5], interleukin-1β (IL-1β) [6], interleukin-6 [7] secreted by M1 ATMs can modify adipocyte function, such as activating inflammatory pathways [8] and impairing insulin action [9]. However, whether cytokines are the only factors for the induction of insulin resistance remains unclear.

Microvesicles (MVs), also known as extracellular vesicles, microparticles, exosomes, or shedding vesicles, are small (30–1000 nm) membrane-bound particles released from eukaryotic cells under normal physiological and pathological conditions. They play a pivotal role in mediating cell-to-cell communication [10]. Mesenchymal stem cell-derived MVs can inhibit *in vitro* islet antigen T cell activation at type 1 diabetes onset [11]. MVs derived from apoptotic endothelial cells [12], activated platelets [13] or monocytes [14] can act as cellular effectors, disseminating pro-inflammatory potential in vascular inflammation, which may contribute to vascular diseases and diabetic cardiovascular complications. Our previous studies [15] and others [16] have further shown that MVs released from inflamed monocytes/macrophages may represent a class of inflammatory factors involved in the inflammatory process associated with metabolic diseases. MVs secreted from monocytes can promote angiogenesis *in vitro* and *in vivo*, which may serve as a novel therapeutic approach for many angiogenesis-related diseases such as cancers and diabetes [17]. Furthermore, growing evidence is demonstrating that monocyte/macrophage-derived MVs play crucial roles in the pathogenesis of inflammatory diseases through up-regulation of proinflammatory mediators, such as nuclear factor (NF)-κB [18, 19]. MVs released by adipocytes play a major role in the crosstalk between adipocytes and macrophages; this crosstalk is now recognized as a major mechanism in adipose tissue inflammation and a key contributor to insulin resistance [20, 21]. The possibility remains that monocyte/macrophage-secreted MVs can inhibit insulin signaling in adipocytes, thereby causing the insulin resistance observed in obese adipose tissue.

In the current study, we characterized MVs shed by *in vitro* polarized THP-1 macrophages with both M1 and M2 phenotypes, and evaluated their ability to influence insulin signaling and glucose uptake in human primary mature adipocytes and primary differentiated adipocytes through activation of NF-κB. The data reported here may expand our knowledge the role of macrophage-derived microvesicles in the crosstalk between macrophages and adipocytes, and provide potential therapeutic targets for obesity-related insulin resistance.

## Materials and methods

### Subjects

Subcutaneous abdominal tissue specimens of human white fat were obtained from nondiabetic females with a BMI of less than 25. Written informed consent was obtained from each patient before the study. All studies were performed with the approval of the Ethics Committee of Nanjing Medical University.

### Isolation, cultivation, and differentiation of preadipocytes

Human primary preadipocyte cultures were prepared as previously described, with minor modifications [22]. Samples of adipose tissue (AT) were washed with PBS supplemented with gentamycin and then digested with collagenase I (1–1.5 g/L, Sigma) for 40–60 min in a shaking water bath at 37 °C. The digest was then filtered through 200 μm mesh and pelleted by centrifuging at 1000 rpm for 5 min. The mature adipocytes were collected and cultivated in adipocyte medium consisting of Dulbecco’s modified Eagle’s medium (DMEM)/F-12, 100 mL/L FBS, 6 × 10^4^ U/L penicillin, and 6 × 10^4^ U/L streptomycin. The pellet containing the stroma-vascular fraction (SVF) was incubated in erythrocyte lysis buffer (155 mmol/L NH_4_Cl, 10 mmol/L KHCO_3_, and 90 μmol/L EDTA) for 10 min at room temperature. For preadipocyte differentiation experiments, SVF cells were collected by centrifugation and, without any filtration step, grown in medium containing DMEM/F-12, 100 mL/L FBS, 15 mmol/L HEPES (pH 7.4), 60 U/mL penicillin, 6 × 10^4^ U/L streptomycin, and 25 mg/L amphotericin B. Cultures were incubated at 37 °C in a 5 % CO_2_, water-saturated atmosphere. SVF cells were grown to 70–80 % confluency and then prepared for differentiation, as described in previous publications [23].

For the first 3 d, cultures were grown in differentiation medium containing DMEM/F-12, 30 mL/L FBS, 15 mmol/L HEPES (pH 7.4), 33 mmol/L biotin, 17 mmol/L pantothenate, 100 nmol/L insulin, 1 mmol/L dexamethasone (DEX), 6 × 10^4^ U/L penicillin, 6 × 10^4^ U/L streptomycin, 25 mg/L Fungizone and 0.25 mmol/L 3-isobutyl-1-methylxanthine (IBMX). Thereafter, the medium was replaced every other day without IBMX. After 10 d, under these culturing conditions, approximately 35 % of the cells exhibited the morphology of mature adipocytes. After 15 d in culture, at least 80 % of the cells contained visual lipid droplets. Lipid accumulation was assessed by staining paraformaldehyde-fixed cells with oil red O and quantified by measuring the optical absorbance at 510 nm. Insulin and other chemicals were from Sigma–Aldrich (St. Louis, MO, USA) or as indicated in the text.

### THP-1 cell culture and stimulation

A human acute monocytic leukemia cell line (THP-1) was purchased from China Cell Culture Center (Shanghai, China) and cultured in standard RPMI 1640 medium supplemented with 10 % fetal bovine serum (FBS; GIBCO) in a 5 % CO_2_, water-saturated atmosphere. For maturation, THP-1 cells were treated with phorbol-12-myristate-13 acetate (PMA) (100 ng/mL, Sigma, St. Louis, MO) for 48 h, after which the medium was aspirated and the cells were washed twice in pre-warmed PBS [24]. For polarization toward the M1 or M2 phenotype, the PMA-differentiated THP-1 macrophages were cultured for 96 h in media containing LPS (100 ng/mL, Calbiochem, Billerica, MA) plus IFN-γ (20 ng/mL, Sigma) or IL-4 (20 ng/mL, Peprotech, Rocky Hill, NJ), respectively [25].

### Analysis morphology and phenotyping of macrophages

For morphological studies, polarized THP-1 macrophages were photographed using differential interference light microscopy.

The cell phenotypes in each condition were determined by quantitation of the expression of cell markers that delineated the M1 and M2 phenotypes by flow cytometry (FCM), as described previously [26]. After the THP-1 cells had been treated as described above, they were scraped, followed by washing with ice-cold PBS and centrifugation. The pellets were stained for 20 min on ice with CD11c-FITC and CD206-PE antibodies (BD Biosciences, San Jose, CA, USA) in PBS containing 1 % FBS. After incubation, the cells were washed with ice-cold PBS three times and resuspended in 200 μL 1 % FBS − PBS. Fluorescence intensities were analyzed by a fluorescence-activated cell sorting (FACS) scan (BD Biosciences, San Jose, CA, USA). All data were analyzed using FlowJo software (Tree star, Ashland, OR, USA).

### Preparation of MVs

MVs were isolated from the THP-1 macrophage culture medium by differential centrifugation, using a previously published technique [15, 27]. Briefly, the supernatant medium was centrifuged at 500 g and then at 1500 g to spin down the mature cells and cell debris. The resultant supernatant was filtered through 0.22 μm film and further centrifuged at 130,000 g for 70 min (all steps were performed at 4 °C), and then the pellet at the bottom of the centrifugation tube was collected (MVs-rich fraction). The BCA method was employed to quantify the total protein content in the MVs. The levels of MVs were determined by measuring the total protein content, which is presented as micrograms of total protein in the MVs.

### Transmission electron microscope (TEM) assay

The MVs were fixed overnight at 4 °C with 2 % glutaraldehyde in 0.1 mol/L phosphate buffer (pH 7.4). The samples were rinsed in PBS buffer and postfixed in 1 % OsO_4_ for 1 h, dehydrated in ethyl alcohol, and embedded in Quetol-812. Ultra-thin sections were etched using saturated sodium metaperiodate, followed by additional etching in 0.1 N HCl for 10 min before observation with a FEI Tecnai T20 transmission electron microscope.

### Fluorescence labeling of MVs and confocal microscopy analysis

Macrophage-like THP-1 (M0 THP-1) cells were labeled with DiI-C16 for 2 h and washed three times with PBS. The cells were resuspended and cultured for 24 h in 1640 medium supplemented with 10 % FBS. The supernatants were then collected and centrifuged to harvest M0 THP-1-secreted MVs (M0 MVs). M0 MVs were re-suspended in PBS and incubated with cultured human preadipocytes. After a fixed time interval (0, 2, or 4 h), preadipocytes were washed, fixed, and observed with confocal microscopy (FV1200; Olympus, Tokyo). The pictures were taken under these conditions: Objective Lens: LUCPLFLN 40×; Scan Mode: XY; Excitation Wavelength: 342 nm for Hoechst and 546 nm for DiI-C16; Image Size: 1024 × 1024 Pixel.

### Quantitative real-time PCR

The qRT-PCR analysis was performed in 96-well plates using SYBR Green-based detection on a StepOnePlus machine (Applied Biosystems, Norwalk, CT, USA). Each 10 μL reaction contained approximately 50 ng of cDNA, 0.3 μM of sense and antisense primers, and 1× QuantiTect SYBR Green SuperMix (TaKaRa, Japan). The plate was then sealed and cycled under the following conditions: 95 °C /10 min; 40 cycles of 95 °C /10 s and 60 °C /45 s. Each reaction was performed in triplicate, using mRNA levels of β-actin for normalization. The sequences of the primers were as follows: CD68 (forward 5′-GCTACATGGCGGTGGAGTACAA-3′; reverse 5′-ATGATGAGAGGCAGCAAGATGG-3′) TNF-α (forward 5′-CCCAGGCAGTCAGATCATCTTCT-3′; reverse 5′-ATGAGGTACAGGCCCTCTGAT-3′); CD206 (forward 5′-CCATGGACAATGCGCGAGCG-3′; reverse 5′-CACCTGTGGCCCAAGACACGT-3′); PPARγ1 (forward 5′-ATTCTGGCCCACCAACTTTG-3′; reverse 5′-TCCATTACGGAGAGATCCACG-3′); PPARγ2 (forward 5′- AGCAAACCCCTATTCCATGCT-3′; reverse 5′- ATCAGTGAAGGAATCGCTTTCTG-3′) and β-actin (forward 5′-CACGAAACTACCTTCAACTCC-3′; reverse 5′- CATACTCCTGCTTGCTGATC-3′).

### Nuclear extract preparation

Nuclear proteins were prepared as previously described [28]. After the indicated treatments, adipocytes were washed three times and resuspended in buffer A [(10 mmol/L KCl, 0.1 mmol/L EDTA, 0.1 mmol/L EGTA,10 mmol/L HEPES; pH 7.9), 1 mmol/L DTT, and 0.5 mmol/L PMSF], followed by centrifugation (10 min, 3000 g at 4 °C). The supernatant was then discarded and the pellet was resuspended in buffer A. After a 15 min incubation on ice, a 1:20 volume of 10 % Nonidet P-40 was added and vortexed. The nuclei were allowed to swell on ice for 15 min and then were pelleted by centrifugation (10 min, 10,000 g at 4 °C). The resulting nuclear extracts were frozen and stored at −80 °C.

### Western blotting

Adipocytes or MVs were lysed with ice-cold lysis buffer containing 50 mmol/L Tris–HCl (pH 7.4), 1 % NP-40, 150 mmol/L NaCl, 1 mmol/L EDTA, 1 mmol/L phenylmethylsulfonyl fluoride, and complete protease inhibitor (1 tablet/10 mL; Roche). After centrifugation at 12,000 rpm for 20 min at 4 °C, the supernatants were collected and protein concentration was determined by the BCA Protein Assay (Thermo Scientific Pierce, Rockford, IL). Proteins (40–60 μg protein/lane) were separated by electrophoresis on 10–12 % polyacrylamide gels, and the bands were subsequently transferred onto polyvinylidene fluoride (PVDF) membranes (Millipore). Membranes were blocked in PBST/5 % nonfat dry milk powder and incubated with antibodies against TSG101 and NF-κB p65 (Santa Cruz Biotechnology, Santa Cruz, CA), GLUT4 (Abcam, Cambridge, UK), phosphorylated Akt (Ser473), Akt and clathrin heavy chain (Cell Signaling Technology, Danvers, MA). Anti-human Akt, clathrin, and histone H3 (Santa Cruz Biotechnology, Santa Cruz, CA) were used as controls.

Plasma membrane proteins from treated adipocytes were prepared using Sigma-Aldrich Proteo-Prep Membrane Extraction Kit [29]. Aliquots containing 80 μg of plasma membrane protein were subjected to 10 % SDS-PAGE, and Western blot analysis was performed as described above.

### Measurement of cytokine release

Human Interleukin (IL)-10 and IL-1β levels were assayed in cell culture supernatants by sandwich enzyme-linked immunosorbent assay (ELISA) using a commercially available ELISA kit (R&D Systems, Minneapolis, MN, USA), according to the manufacturer’s instructions.

### Glucose uptake measurement by 2-NBDG

The glucose uptake assay with the 2-NBDG screening system was performed as previously described, with some modifications [30]. Briefly, human primary preadipocytes were first differentiated in 96-well plates and then cultured for 24 h in DMEM/F12 medium containing 100 μg of the indicated MVs. After 24 h, adipocytes were washed in Krebs-Ringer phosphate (KRP) buffer (pH 7.4) (136 mmol/L NaCl, 4.5 mmol/L KCl, 1.25 mmol/L CaCl_2_, 1.25 mmol/L MgCl_2_, 0.6 mmol/L Na_2_HPO_4_, 0.4 mmol/L NaH_2_PO_4_, 10 mmol/L HEPES, and 0.1 % BSA), and incubated at 37 °C for 20 min in 50 μL KRP with the given concentration of insulin. The cells were then sequentially cultured with or without 10 mM 2-NBDG in 50 μL KRP for a further 1 h. The fluorescence intensity of 2-NBDG was recorded using an F-7000 fluorescence spectrophotometer (Hitachi, Tokyo, Japan). False positives were ruled out by treating with MVs in the absence of 2-NBDG and using these measurements as the background. The relative fluorescence intensities minus the background levels were used for data analysis.

### MTT assay

The preadipocytes were seeded at a density of 1 × 10^4^ cells/well in 96-well culture plates. Then, the cells were treated with BAY 11–7082 at the various concentrations (2.5, 5, 10 and 20 μM) for 24 h. After completion of the treatment, the cells were incubated with MTT (Sigma-Aldrich, USA) solution (2 mg/mL) for 4 h at 37 °C. The supernatants were aspirated, DMSO was added, and the plates were agitated to dissolve the crystal product. Absorbance was measured at 490 nm (570 nm as a reference) using a StatFAX303 plate reader.

### Data analysis

The western blot images are representative of at least three independent experiments. The values for the FCM, qRT-PCR, ELISA, 2-NBDG and MTT assays were from three independent experiments performed in triplicate. The data are expressed as the mean ± SEM of three independent experiments. Statistical significance was determined using Student’s *t*-test and set as *P* < 0.05.

## Results

### Characterization of polarized macrophages

**Fig. 1:**
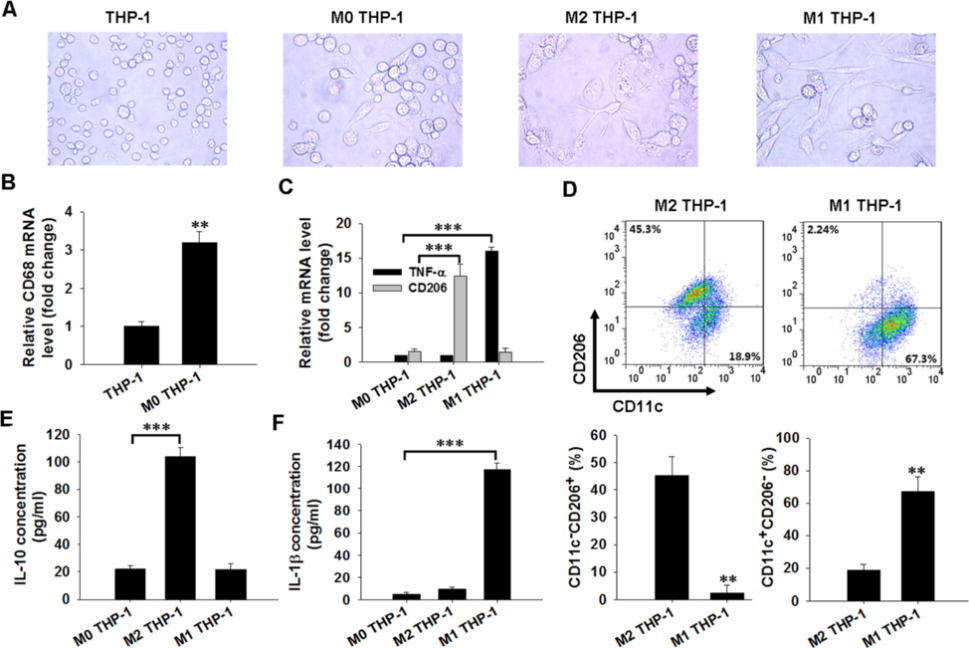
Detection of macrophage polarization. **a**. Morphology of THP-1 monocytes, M0 THP-1, M2 THP-1, and M1 THP-1 cells were examined by light microscopy (100×). **b**, **c**. Phenotypic gene expression in M0 THP-1, M1 THP-1, and M2 THP-1 cells was analyzed by qRT-PCR. **d**. Expression of CD11c and CD206 was determined by flow cytometry. Quantification of the flow cytometry assay was analyzed using FlowJo software, and statistical analysis is presented below. **e**, **f**. IL-10 (**e**) and IL-1β level (**f**) in the cell culture supernatant were analyzed by ELISA. **P < 0.01 indicates a significant difference compared with the corresponding vehicle groups. ***P < 0.001 indicates a significant increase when compared with the indicated conditions

Differentiated THP-1 monocytes have been widely used as an *in vitro* model of human macrophages. Classically (M1) or alternatively (M2) activated macrophage populations were derived from THP-1 cells by conducting serial dose and time optimization experiments. As shown in Fig. 1a, light microscopy showed that monocyte-like THP-1 cells (THP-1) displayed a round shape and a nonadherent pattern, while macrophage-like THP-1 cells (M0, M2 and M1 THP-1) were adherent, with the typical flat, amoeboid-shaped, elongated, and branching macrophage morphology. The macrophage-like THP-1 (M0 THP-1) phenotype was also illustrated by the increase in CD68 mRNA revealed by the qRT-PCR assay (Fig. 1b). Most importantly, another feature of M2 and M1 THP-1 macrophage polarization was enhanced marker gene and protein expression, as confirmed by qRT-PCR and FCM quantification assays. Fig. 1c shows that, in contrast to M0 THP-1 cells, M1 THP-1 cells preferentially expressed pro-inflammatory genes (i.e., TNF-α), whereas M2 THP-1 cells mainly expressed anti-inflammatory genes (e.g., the macrophage mannose receptor, CD206). FCM quantification of M1 THP-1 cells also showed an increased level of CD11c, which is crucial for the activation of M1 macrophage, and a decreased level of CD206 as compared with M2 THP-1 cells (Fig. 1d). The M1 and M2 THP-1 phenotypes were confirmed by characterization of secreted cytokines. As expected, M1 THP-1 cells significantly enhanced the secretion of IL-1β, a master proinflammatory cytokine (Fig. 1f), whereas M2 THP-1 cells mostly secreted IL-10 into the medium (Fig. 1e). Collectively, these results suggest that THP-1 cells were successfully polarized into M1-like or M2-like macrophages after PMA, LPS plus IFN-γ, or IL-4 stimulation.

### Internalization of macrophage-derived MVs into adipocytes

**Fig. 2:**
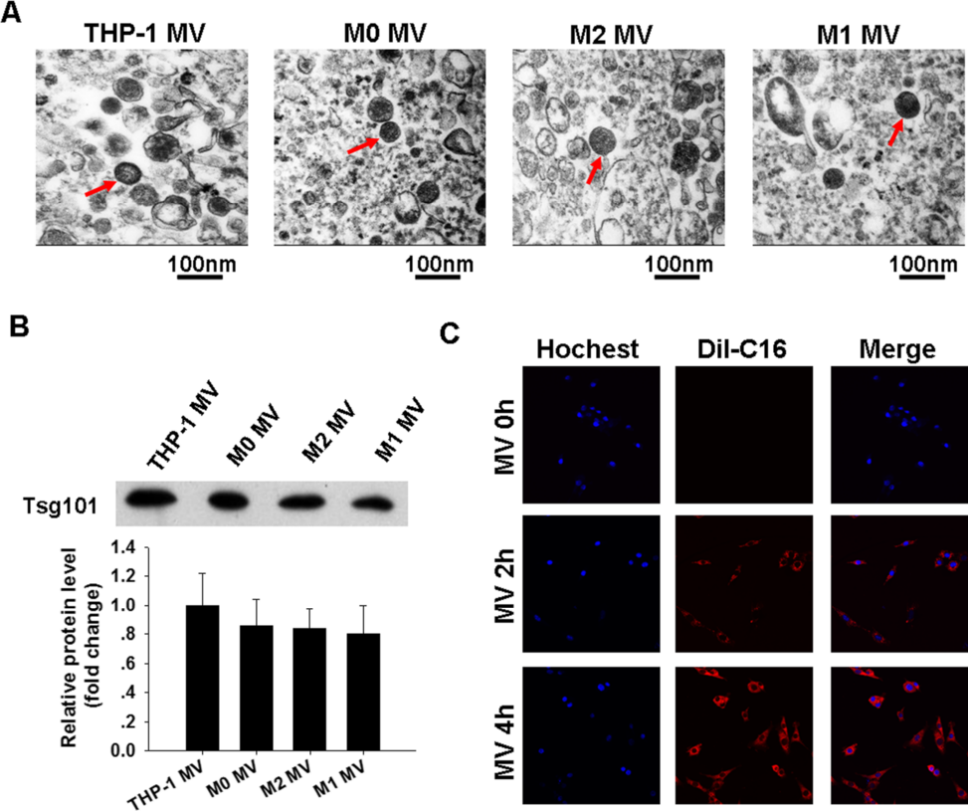
Internalization of macrophage-derived MVs into adipocytes. **a**. Micrographs of MVs isolated from culture medium of THP-1 monocytes and polarized THP-1 macrophages examined by TEM. These particles are highlighted by red arrows. Scale bars are all equal to 100 nm. **b**. Equal amounts of proteins from indicated MVs were analyzed using western blotting for exosome-enriched protein TSG10. Western blot images were analyzed by Bandscan software, and statistical analysis is presented below. **c**. Internalization of fluorescently labeled M0 MVs into adipocytes was analyzed by confocal microscopy (400×)

Previously, studies by our laboratory [15] and others [31] have demonstrated that MVs derived from monocyte-like THP-1 cells can enter into recipient cells. Accordingly, we tested whether macrophage-like THP-1-secreted MVs (e.g. M0 MVs) can enter the adipocytes. We first characterized the MVs shed by macrophage-like THP-1 cells by TEM and western blot assays. As shown in Fig. 2a (indicated by red arrows), the isolated macrophage-like THP-1 MVs were vesicles 30–100 nm in diameter, and each of these vesicles was surrounded by a double-layer membrane, which was consistent with monocyte-like THP-1 cell-derived MVs (THP-1 MVs). Meanwhile, the ubiquitin-binding protein and exosome marker TSG101 [32] was highly expressed in the MVs (Fig. 2b). Interestingly, these vesicles were also taken up by adipocytes. Fluorescence-labeled M0 MVs were incubated with unstained adipocytes. As depicted in Fig. 2c, labeled MVs rapidly entered the recipient cells; this uptake was an active process that depended on the incubation time. This experiment clearly demonstrated that macrophage-secreted MVs could be effectively internalized into adipocytes.

### Effect of macrophage-derived MVs on insulin signaling in human adipocytes

**Fig. 3:**
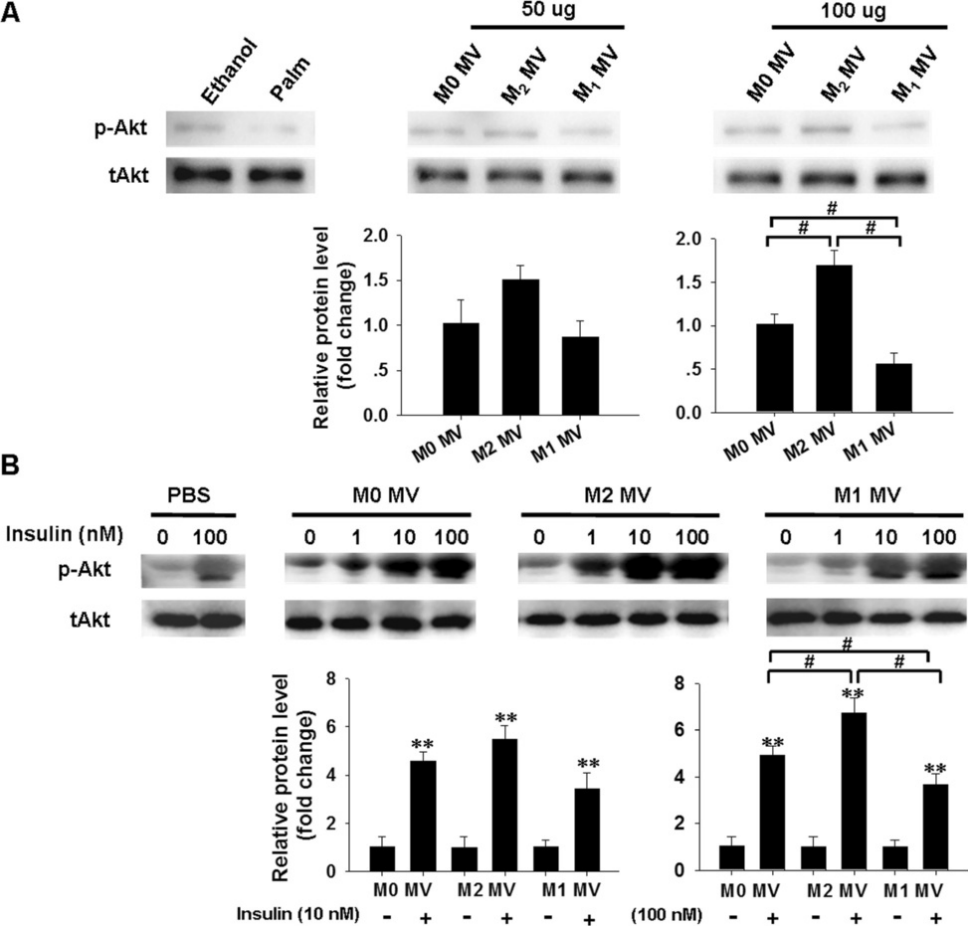
Effects of macrophage-derived MVs on insulin-stimulated Akt phosphorylation in human mature adipocytes. **a**. The level of pAkt in human mature adipocytes after treatment with various concentrations of M0 MVs, M1 MVs, and M2 MVs was detected by western blot assays. Ethanol treatment was used as the negative control, and the palm group was the positive control. Western blot images were analyzed using Bandscan software, and statistical analysis is presented below. **b**. Levels of insulin-stimulated pAkt protein in adipocytes after treatment with indicated MVs were determined by western blotting. PBS treatment was used as the basal control. Quantification of the pAkt protein levels is shown below. **P < 0.01 indicates a significant enhancement compared with the corresponding vehicle control. #*P* < 0.05 indicates a significant difference when compared with the indicated conditions

The effects of MVs [derived from M1 THP-1 (M1 MVs) and M2 THP-1 (M2 MVs) macrophages] on insulin signaling were examined by measuring the phosphorylation of the serine/threonine kinase Akt (pAkt), a central player in the insulin signaling pathway. Human primary mature adipocytes were used as they represent a primary cell type for insulin action and the development of insulin resistance in obesity. These adipocytes were isolated and cultivated in complete DMEM/F-12 medium (Additional file 1: Figure S1A), followed by a 24 h treatment with various concentrations of M1 MVs and M2 MVs, and a subsequent stimulation with 100 nM insulin for 20 min. M0 MVs was employed as a normal control. Fig. 3a shows that treatment with 100 μg M1 MVs significantly reduced the level of pAkt, whereas treatment with M2 MVs applied at the same concentration mainly induced pAkt expression, when compared with M0 MVs-treated cells. Thus, 100 μg MVs were used in all further experiments in this study.

The impact of MVs on insulin signaling was investigated by pretreating human subcutaneous adipocytes with the indicated MVs for 24 h, and then incubating the cells with various concentrations of insulin for 20 min, followed by analysis of pAkt expression. Fig. 3b shows that the level of pAkt was significantly increased after insulin stimulation, in a dose-dependent manner. Importantly, M1 MVs consistently induced a marked reduction of pAkt level in response to 100 nM insulin (Fig. 3b, lane 14), in contrast to the higher pAkt level observed following M2 MVs treatment (Fig. 3b, lane 10), when compared to the M0 MVs-treated control.

**Fig. 4:**
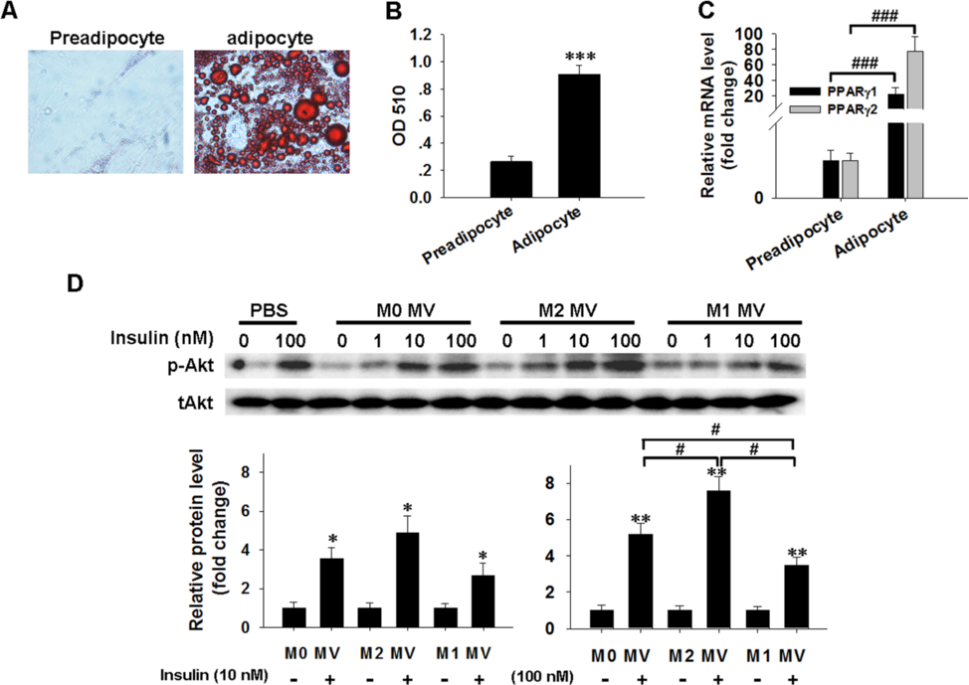
Roles of macrophage-derived MVs in insulin-stimulated pAkt levels in human differentiated adipocytes. **a**. Human differentiated adipocytes were fixed and stained with oil red O for lipid accumulation. (200×). **b**. The optical absorbance of lipid accumulation was measured at 510 nm. **c**. The mRNA levels of PPARγ1 and PPARγ2 in preadipocytes and adipocytes were analyzed by qRT-PCR. **d**. Protein levels of pAkt in human differentiated adipocytes were detected by western blotting. Adipocytes were pretreated with various reagents for 24 h, and then the cells were stimulated with 0, 1, 10, or 100 nM insulin for 20 min, lysed with lysis buffer, and prepared for the immunoblotting assay. Total Akt (tAkt) was used as a loading control. Western blot images were analyzed using Bandscan software, and statistical analysis is presented below. *P < 0.05, **P < 0.01 and ***P < 0.001 indicate a significant up-regulation compared with the corresponding vehicle groups. #*P* < 0.05 and ###*P* < 0.001 indicate significant difference when compared with indicated conditions

We also assessed the role of M1 MVs and M2 MVs on insulin-stimulated pAkt in differentiated human adipocytes. A homogenous adipocyte fraction was obtained by isolating human primary pre-adipocytes (Additional file 1: Figure S1B) and differentiating these into adipocytes; this differentiation was morphologically monitored (Fig. 4a, b). As expected, qRT-PCR assays also confirmed a significant up-regulation of two mRNA markers of adipocytes [33]; namely, peroxisome proliferator activated-receptor γ1 (PPARγ1, fold-changes control = 20, *p* < 0.001) and PPARγ2 (fold-changes control = 80, *p* < 0.001), which are key regulators of adipogenesis. Both were highly expressed in adipocytes compared to pre-adipocytes (Fig. 4c). The differentiated adipocytes were then pre-treated with the indicated MVs followed by stimulation by various concentrations of insulin. Fig. 4d shows that stimulation with 100 nM insulin and M1 MVs inhibited of Akt phosphorylation (lane 14), whereas M2 MVs treatment reversed this response in treated adipocytes (lane 10), compared with M0 MVs-treated control (lane 6). Taken together, these results suggest that macrophage-derived MVs may functionally regulate insulin signaling and that M1 MVs may play a critical role in decreasing insulin signal transduction in adipocytes, whereas M2 MVs have the opposite effect.

### Effect of macrophage-derived MVs on insulin-induced glucose uptake in human adipocytes

**Fig. 5:**
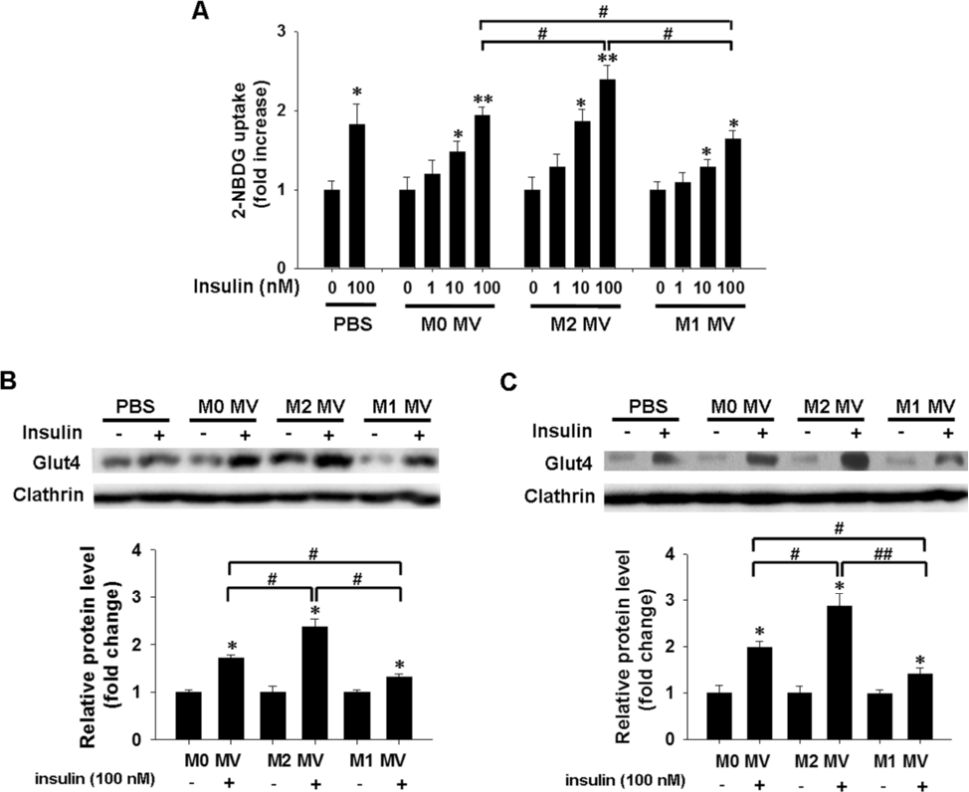
Effects of macrophage-derived MVs on glucose uptake and GLUT4 translocation in human adipocytes. **a**. The insulin-stimulated glucose uptake of treated differentiated adipocytes was measured by 2-NBDG assay. The relative fluorescence intensities minus the background levels were used for statistical analysis. **b**, **c**. The level of GLUT4 in plasma member (PM) fractions of human mature adipocytes (**b**) and differentiated adipocytes (**c**) was determined by western blotting. Adipocytes were treated with the indicated MVs for 24 h, and were then treated with or without 100 nM insulin for a further 20 min. Cells were homogenized, and PM fractions were obtained and subjected to immunoblotting with antibodies against GLUT4, or clathrin heavy chain, as indicated. Western blot images were analyzed using Bandscan software, and statistical analysis is presented below. *P < 0.05 and **P < 0.01 indicate a significant augmentation compared with the corresponding vehicle treated control. #*P* < 0.05 and ##*P* < 0.01 indicate significant a difference when compared with the indicated conditions

The effects of macrophage-derived MVs on insulin sensitivity were further assessed by assaying 2-NBDG uptake in terminally differentiated adipocytes following treatment with indicated MVs for 24 h, followed by stimulation with various concentrations of insulin for 20 min. As illustrated in Fig. 5a, insulin induced a dose-dependent increase in glucose uptake in human differentiated adipocytes. Compared with the M0 MVs treated control, M1 MVs markedly decreased glucose uptake into treated adipocytes, whereas M2 MVs clearly enhanced this uptake following 100 nM insulin stimulation (Fig. 5a).

The possibility that the reduction (or enhancement) of glucose uptake into adipocytes could be explained by M1 MVs/M2 MVs-induced changes in the amounts of glucose transporter protein at the cell surface was addressed by measuring the protein level of GLUT4, one of the predominant glucose transporter isoforms expressed in adipocytes. GLUT4 levels were measured in plasma membrane (PM) fractions in the basal state or after treatment with M1 MVs or M2 MVs, following incubation with or without 100 nM insulin. PM fractions prepared from basal (PBS and M0 MVs-treated group), M1 MVs-treated and M2 MVs-treated adipocytes contained comparable amounts of clathrin [34]. In the absence of insulin, the PMs of human mature adipocytes (Fig. 5b, lane 1) and differentiated adipocytes (Fig. 5c, lane 1) contained only low amounts of GLUT4. Insulin exposure (100 nM) substantially increased the GLUT4 content in the PMs (Fig. 5b and c, lane 2), in line with previously reported findings [35]. Notably, M2 MVs induced a significant increase in the GLUT4 protein of the PMs (Fig. 5b and c, lane 6), whereas M1 MVs were less effective than M2 MVs at inducing PM translocation of GLUT4 (Fig. 5b and c, lane 8), when compared to the M0 MVs-treated group (Fig. 5b and c, lane 4). The M1 MVs-induced decrease in glucose uptake was associated with the presence of only half the amount of GLUT4 in the PM fraction, whereas M2 MVs increased GLUT4 levels in the PM. Collectively, these results indicate that, compared with M2 MVs, M1 MVs induce a marked reduction of glucose uptake activity by decreasing the translocation of GLUT4 to the adipocyte PM.

### Effect of macrophage-derived MVs on insulin-stimulated activation of NF-κB in human adipocytes

**Fig. 6:**
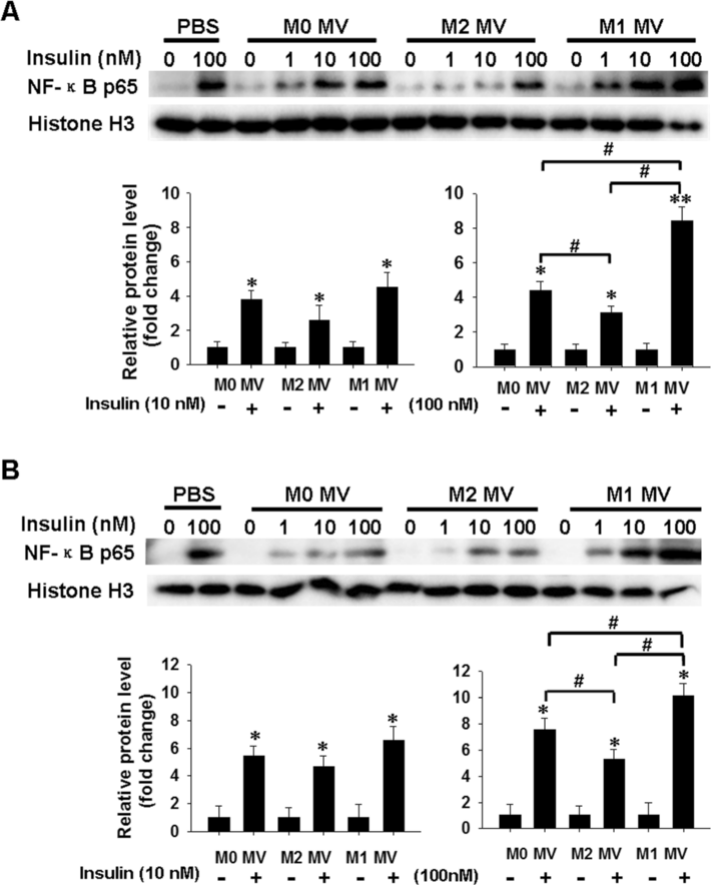
Effect of macrophage-derived MVs on insulin-stimulated activation of NF-κB in human adipocytes. **a**, **b**. Relative protein levels of nuclear NF-κB p65 in human mature adipocytes (**a**) and differentiated cells (**b**) were analyzed by western blotting. Human adipocytes were treated with indicated MVs for 24 h, and then incubated with 0, 1, 10, or 100 nM of insulin for 20 min. NF-κB p65 and histone H3 levels in nuclear lysates were determined by immunoblotting. Quantification of NF-κB p65 protein level is shown below. Western blot images were analyzed using Bandscan software, and statistical analysis is presented below. *P < 0.05 and **P < 0.01 indicate a significant enhancement compared with the corresponding vehicle-treated control. #*P* < 0.05 and ##*P* < 0.01 indicate a significant difference when compared with the indicated conditions

Nuclear factor kappa B (NF-κB) is a transcription factor that is thought to play a central role in obesity-associated inflammation and insulin resistance [36]. In a quiescent state, NF-κB is confined to the cytoplasm by the inhibitor of κB-α (IκBα). The activated inhibitor of the kappa B kinase complex (IKK) phosphorylates IκBα, which leads to IκBα polyubiquitination and degradation, thereby freeing NF-κB to translocate into the nucleus, where it induces target gene expression [37]. The possibility that M1 MVs and M2 MVs might mediate the effect of NF-κB activation on insulin signaling and glucose uptake in adipocytes was evaluated by analyzing the levels of NF-κB p65 in nuclei of human mature adipocytes and differentiated adipocytes, following treatment with indicated MVs and insulin. Fig. 6a and b show that stimulation by 100 nM insulin strongly increased the basal level of nuclear NF-κB p65 (PBS group, lane 2) in mature (Fig. 6a) and differentiated adipocytes (Fig. 6b), in agreement with previous findings showing that insulin activates NF-κB in mammalian cells [38]. The expression of NF-κB was increased upon treatment with insulin in a dose-dependent manner. Compared with M0 MVs (Fig. 6a and b, lane 6), the level of NF-κB p65 in nuclei was significantly increased after M1 MVs treatment (Fig. 6a and b, lane 14), whereas the nuclear marker protein, histone H3, was not affected by M1 MVs treatment. In contrast, incubation with M2 MVs strongly decreased the level of nuclear NF-κB p65 in adipocytes (Fig. 6ba and , lane 10).

**Fig. 7:**
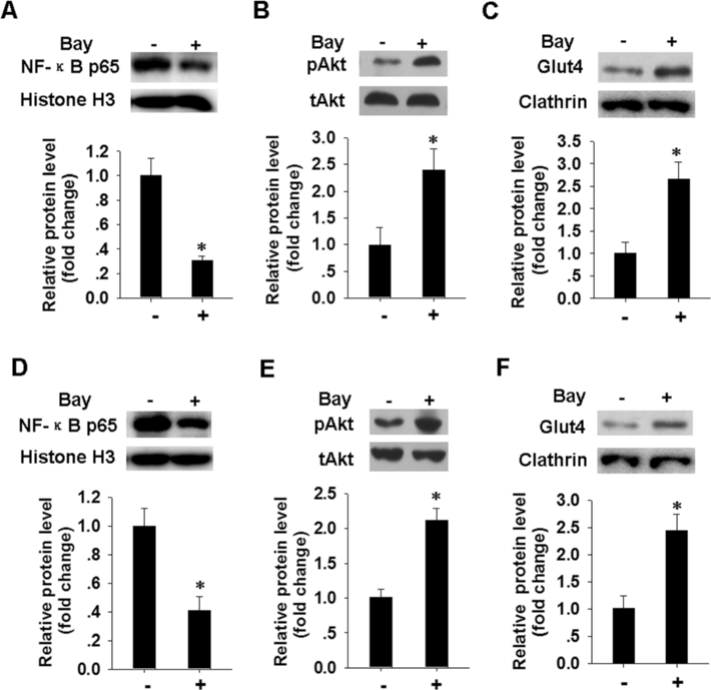
Blocking of NF-κB reverse the inhibitory effect of M1 MVs on pAkt level and GLUT4 translocation in human adipocytes. **a**, **d**. Expression level of nuclear NF-κB p65 in human mature adipocytes (**a**) and differentiated cells (**d**) were analyzed by western blotting. Adipocytes were pretreated with or without NF-κB specific inhibitor (Bay, 10 μM) for 2 h, then incubated with M1 MVs for 24 h. Nuclear lysates were analyzed for NF-κB p65 and histone H3 level after insulin stimulation for a further 20 min. Quantification of NF-κB p65 protein level is presented below. **b**, **e**. Protein level of pAkt in human mature adipocytes (**b**) and differentiated adipocytes (**e**), after treatment as in **a**. Western blot images were analyzed using Bandscan software, and statistical analysis is presented below. **c**, **f**. The levels of GLUT4 in plasma membrane (PM) fractions of human mature adipocytes (**c**) and differentiated adipocytes (**f**) were determined by western blotting. Cells were treated as in A, and PM fractions were obtained and subjected to immunoblotting with the indicated antibodies. Quantification of the GLUT4 protein level is shown below. *P < 0.05 indicates a significant difference compared with corresponding vehicle-treated control

The effect of M1 MVs on NF-κB activation and triggering of insulin resistance in adipocytes was further evaluated by treatment with BAY 11–7085, a reversible small molecule inhibitor of IκBα phosphorylation (Bay hereafter) [39]. Fig. 7a and d show that a 2 h prestimulation of mature adipocytes (Fig. 7a) and differentiated adipocytes (Fig. 7d) with 10 μM Bay (a concentration shown to be effective in reducing p65 activity) [40] did not affect adipocyte viability, as assessed by the MTT assay (Additional file 2: Figure S2). Subsequent treatment with M1 MVs resulted in a pronounced reduction in the level of nuclear NF-κB in the Bay treated cells compared to cells not treated with Bay. The same treatment condition also significantly enhanced the expression of pAkt (Fig. 7b, e) and GLUT4 translocation (Fig. 7c, f). Taken together, our results suggest that M1 MVs-mediated changes in insulin signaling and glucose uptake may be dependent on their modulation of NF-κB activity.

## Discussion

During the progression of obesity, an accumulation of macrophages and other immune cells occurs in adipose tissue [41]. The resulting reinforcement of macrophage-adipocyte crosstalk in obesity affects adipose tissue biology; however, the molecular mechanisms and the key mediators, particularly those in human adipose tissue, are still poorly understood. This work used *in vitro* models of human macrophages (a macrophage-like THP-1 cell line) and primary adipocytes to investigate the role of polarized macrophage-derived MVs on insulin signal transduction and glucose uptake in human adipocytes. We clearly demonstrated that MVs derived from inflamed macrophages (M1 MVs) decreased the insulin-stimulated phosphorylation of Akt protein through enhancement of NF-κB activation and they blocked glucose uptake by blunting signaling responses that lead to glucose transporter (GLUT4) translocation to the cell surface. By contrast, MVs secretion by anti-inflammatory macrophages (M2 MVs) significantly induced pAkt expression, which evoked an increase in glucose transport into the adipocytes. This implicates M1 MVs as important factors for aggravating insulin resistance in obese adipose tissue, whereas M2 MVs may reverse this effect. This possibility could be important for developing effective therapeutic targets.

Our previous study demonstrated that monocytic THP-1 (myeloid leukemic cell line)-secreted MVs could communicate with a variety cell types, such as human microvascular endothelial cells [15] and tumor-associated macrophages [42], suggesting that these MVs may have a widespread impact. Therefore, in this study, we employed THP-1-derived macrophages as a cell model to detect the effect of MVs secreted by macrophage-like THP-1 cells on human adipocytes. We found that MVs shed by monocyte-like THP-1 and macrophage-like THP-1 cells have a uniform vesicular structure and a greater expression of the exosomal marker protein TSG101. The MVs derived from macrophages are also capable of internalizing into adipocytes in a time-dependent manner, as determined using labeled MVs; this uptake is essential for mediation of adipocyte responses independently of cytokines.

Identification of the key factors that mediate the detrimental effects of macrophages on adipocytes is crucial for the development of effective therapeutic targets. Macrophage-derived cytokines have been implicated as key regulators in the transformation of obesity-associated inflammation into insulin resistance in rodents and humans. For example, Medina et al. reported that macrophage-derived TNF-α played a key role in impairing Akt-dependent insulin signaling in adipocytes by decreasing Akt levels [43]. Lagathu et al. demonstrated that IL-1β, released by macrophages, mediates the effect on insulin signaling transduction and proinflammatory response in human primary adipocytes through effects on the insulin signaling pathway [6]. IL-6-secreted by macrophages also impaired insulin signaling and action in 3 T3-L1 adipocytes through effects on gene transcription of IRS-1, GLUT4, and PPARγ [7, 44].

However, the onset of insulin resistance presents an interesting challenge because of its multifactorial character. In addition to secreted cytokines, other factors in adipose tissue may contribute to the adipocyte-macrophage interaction. In the present study, we showed that, as is seen with cytokines, M1 MVs (inflamed macrophage-secreted MVs) also substantially inhibited the insulin signaling pathway in human adipocytes. Akt phosphorylation is a key enzyme modification involved in insulin signaling [45] and the level of pAkt was significantly increased with insulin stimulation in a dose-dependent manner. When compared with M0 MVs, M1 MVs significantly decreased Akt signaling in response to insulin (100 nM) stimulation in both human mature and differentiated adipocytes, whereas M2 MVs dramatically increased pAkt level and enhanced Akt signaling. These findings suggest that M1 MVs may potentially inhibit Akt phosphorylation, and may serve as a potential mediator for the inhibition of insulin signaling by macrophage activation.

Glucose homeostasis is determined by glucose production and utilization in the insulin-sensitive organs and tissues, including muscle, liver, and adipose tissue. Glucose uptake in adipose tissue plays a critical role in the body glucose control [46], which is demonstrated by the selective depletion of GLUT4 in adipose tissues of mice [47]. In this study, we observed insulin stimulation of glucose uptake in adipocytes in a dose-dependent manner in control or MVs-treated conditions. In the presence of 100 nM insulin, M1 MVs significantly decreased glucose uptake by blocking GLUT4 (insulin-dependent glucose transporter) translocation in adipocytes, as compared to M0 MVs treated adipocytes, while treatment with M2 MVs gave an opposite response. The results shown in Figs. 3 and 4 indicate that the suppression of protein phosphorylation and glucose transport resulted mainly from the interaction of MVs and adipocytes, where MVs derived from inflamed macrophages blunted insulin action in adipocytes. Therefore, MVs secreted by inflammation-activated macrophages may serve as potential inhibitors of insulin signaling and lead to insulin resistance in adipocytes.

The role played by MVs in insulin signaling is complex because of the heterogeneous and unknown contents (proteins, RNAs, lipid, etc.) of MVs that could be providing the signal(s) to modulate the function of recipient cells. The mechanisms by which M1 MVs can interfere with insulin signaling and glucose uptake in adipocytes are currently unknown. The nuclear IKK/NF-κB [36] signaling pathway has been well characterized and mediates the inflammatory responses that further exacerbate insulin sensitivity and glucose/lipid homeostasis [48]. We explored the possible mechanism of MVs action by examining the nuclear translocation of NF-κB, a hallmark of NF-κB activation, in MVs-treated adipocytes. We clearly demonstrated the involvement of NF-κB activation in the observed decrease in pAkt expression and GLUT4 translocation elicited by M1 MVs in adipocytes.

First, we found that insulin induced the activation of NF-κB in a dose-dependent manner, in accordance with previous studies [38]. Second, compared with M2 MVs, M1 MVs showed pro-inflammatory activities evoked by enhancement of NF-κB nuclear translocation. Treatment with Bay, which inhibits NF-κB by prevention of entry of the p65 subunit into the nucleus, resulted in marked down-regulation of NF-κB p65 translocation in M1 MVs-treated cells. Third, we found that adipocytes pretreated with Bay had clearly increased levels of pAkt and GLUT4 translocation following M1 MVs treatment.

NF-κB has no known phosphatase activity, so NF-κB might not have a direct action on the insulin-signaling pathway. The role of NF-κB in insulin action needs further research. We speculate the MVs derived from M1 macrophages may form inflammasomes that induce the inflammation of fat cells. The NF-κB-dependent inflammatory pathway is then activated and this leads to disruption of insulin signaling and glucose transport. The actual molecular signal packaged inside the MVs remains to be determined in future research.

## Conclusions

The results of the current study indicate that MVs derived from inflamed macrophages (M1 MVs) play a role in reducing insulin signal transduction and in decreasing insulin-stimulated glucose uptake in human adipocytes by activation of NF-κB. This may represent a novel mechanism of insulin resistance in adipose tissue, which makes MVs attractive candidates for insulin resistance therapy.

## Additional files

Additional file 1: Figure S1. Light microscopy images of adipocytes. A. Image of human primary mature adipocytes isolated from adipocyte tissue was examined by light microscopy (100×). B. Image of human preadipocytes isolated and cultivated for 3 d was obtained by light microscopy (100×). Additional file 2: Figure S2. The survival rate of pre-adipocytes was determined by MTT after treatment with indicated concentration of BAY 11-7082 (Bay).

## Abbreviations

ATMs: Adipose tissue macrophages
M1 ATMs: Classically activated or pro-inflammatory ATMs
M2 ATMs: Alternatively activated or anti-inflammatory ATMs
MVs: Microvesicles
NF-κB: Nuclear factor kappa B
pAkt: Phosphorylation of the serine/threonine kinase Akt
THP-1 MVs: Monocyte-like THP-1 cell-derived MVs
M0 MVs: Macrophage-like THP-1-secreted MVs
M1 MVs: MVs derived from M1 THP-1
M2 MVs: MVs derived from M2 THP-1
PPARγ: Peroxisome proliferator activated-receptor γ
GLUT4: Glucose transporter 4
