# Supplementary material for: Inflamed macrophage microvesicles induce insulin resistance in human adipocytes
Source: Nutr Metab (Lond). 2015 Jun 6;12:21. doi: 10.1186/s12986-015-0016-3 (PMC4462080; doi:10.1186/s12986-015-0016-3)
Supplement: Additional file 1: Figure S1. — Light microscopy images of adipocytes. A. Image of human primary mature adipocytes isolated from adipocyte tissue was examined by light microscopy (100×). B. Image of human preadipocytes isolated and cultivated for 3 d was obtained by light microscopy (100×). [file 12986_2015_16_MOESM1_ESM.doc]

Additional file 1.

Figure S1. Light microscopy images of adipocytes.


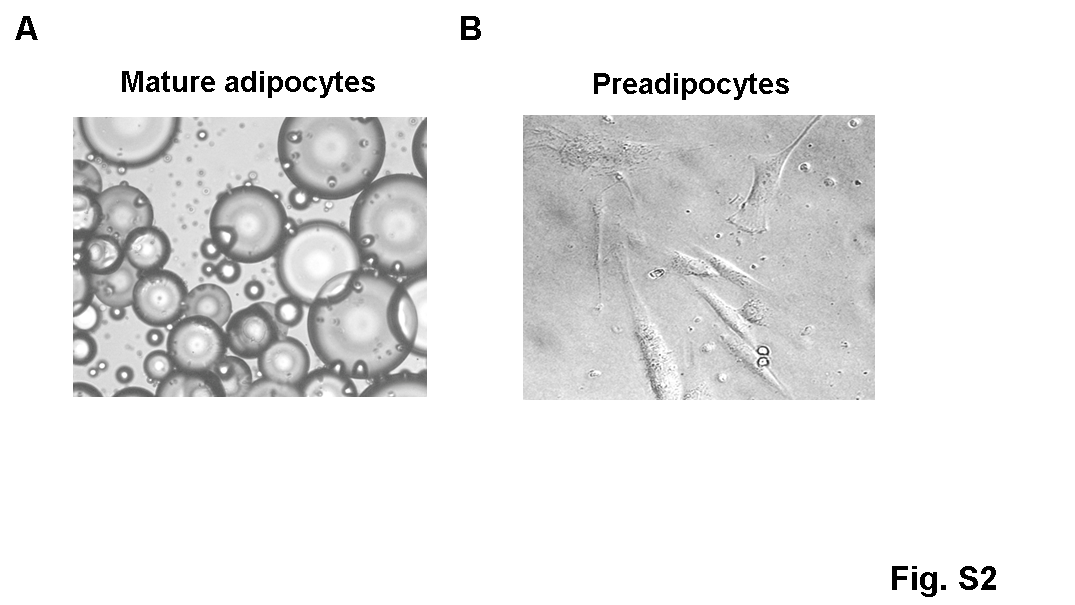


A. Image of human primary mature adipocytes isolated from adipocyte tissue was examined by light microscopy(100×).

B. Image of human preadipocytes isolated and cultivated for 3 d was obtained by light microscopy(100×).
