# Supplementary material for: Inflamed macrophage microvesicles induce insulin resistance in human adipocytes
Source: Nutr Metab (Lond). 2015 Jun 6;12:21. doi: 10.1186/s12986-015-0016-3 (PMC4462080; doi:10.1186/s12986-015-0016-3)

Additional file 2.

Figure S2. The survival rate of pre-adipocytes was determined by MTT after treatment with indicated concentration of BAY 11-7082 (Bay).


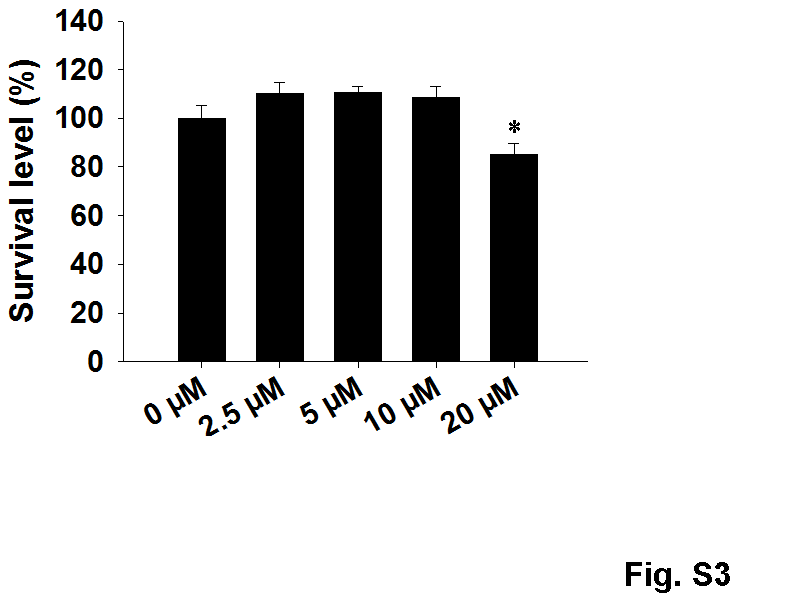

Supplement: Additional file 2: Figure S2. — The survival rate of pre-adipocytes was determined by MTT after treatment with indicated concentration of BAY 11-7082 (Bay). [file 12986_2015_16_MOESM2_ESM.doc]
